# Community mobilisation to prevent violence against women and girls in eastern India through participatory learning and action with women’s groups facilitated by accredited social health activists: a before-and-after pilot study

**DOI:** 10.1186/s12914-020-00224-0

**Published:** 2020-03-25

**Authors:** Nirmala Nair, Nayreen Daruwalla, David Osrin, Suchitra Rath, Sumitra Gagrai, Rebati Sahu, Hemanta Pradhan, Megha De, Gauri Ambavkar, Nibha Das, G. Pramila Dungdung, Damini Mohan, Bahadur Munda, Vijay Singh, Prasanta Tripathy, Audrey Prost

**Affiliations:** 1 Ekjut, Chakradharpur, Jharkhand India; 2 Society for Nutrition, Education and Health Action, Mumbai, Maharashtra India; 3 Institute for Global Health, University College London, London, UK

**Keywords:** Violence against women and girls, India, Community mobilisation, Indigenous communities, Participatory learning and action

## Abstract

**Background:**

Almost one in three married Indian women have ever experienced physical, sexual, or emotional violence from husbands in their lifetime. We aimed to investigate the preliminary effects of community mobilisation through participatory learning and action groups facilitated by Accredited Social Health Activists (ASHAs), coupled with access to counselling, to prevent violence against women and girls in Jharkhand, eastern India.

**Methods:**

We piloted a cycle of 16 participatory learning and action meetings with women’s groups facilitated by ASHAs in rural Jharkhand. Participants identified common forms of violence against women and girls, prioritised the ones they wanted to address, developed locally feasible strategies to address them, implemented the strategies, and evaluated the process. We also trained two counsellors and two ASHA supervisors to support survivors, and gave ASHAs information about legal, health, and police services. We did a before-and-after pilot study involving baseline and endline surveys with group members to estimate preliminary effects of these activities on the acceptability of violence, prevalence of past year emotional and physical violence, and help-seeking.

**Results:**

ASHAs successfully conducted monthly participatory learning and action meetings with 39 women’s groups in 22 villages of West Singhbhum district, Jharkhand, between June 2016 and September 2017. We interviewed 59% (679/1149) of women registered with groups at baseline, and 63% (861/1371) at endline. More women reported that violence was unacceptable in all seven scenarios presented to them at endline compared to baseline (adjusted Odds Ratio [aOR]: 1.87, 95%: 1.39–2.52). Fewer women reported experiencing emotional violence from their husbands in the last 12 months (aOR: 0.55, 95% CI: 0.43–0.71), and more sought help if it occurred (aOR: 2.19, 95% CI: 1.51–3.17). In addition, fewer women reported experiencing emotional or physical violence from family members other than their husbands in the last 12 months (aOR: 0.41, 95% CI: 0.32–0.53, and aOR: 0.36, 95% CI: 0.26–0.50, respectively).

**Conclusion:**

Combining participatory learning and action meetings facilitated by ASHAs with access to counselling was an acceptable strategy to address violence against women and girls in rural communities of Jharkhand. The approach warrants further implementation and evaluation as part of a comprehensive response to violence.

## Background

Around the world, at least one in three women is forced into sex, beaten, or otherwise harmed in her lifetime [1]. In addition, millions of girls are exposed to physical, emotional, and sexual violence [2]. Survivors often experience severe physical and psychosocial sequelae [3, 4].

According to India’s National Family Health Survey (NFHS-4, 2015–6), 31% of married women in India have ever experienced physical, sexual or emotional violence from their husbands, or spousal violence [5]. Physical violence is the most common form of spousal violence (27%), followed by emotional (13%) and sexual violence (6%) [5]. Violence perpetrated by other family members is rarely measured, but some studies suggest that abuse from in-laws is common [6, 7]. Girls are not spared: at least 16% of girls aged 15–19 report being physically or sexually abused, and many more suffer violence earlier in life [5]. Women and girls with multiple intersecting socio-economic vulnerabilities – the poorest, those from Scheduled Caste or Schedule Tribe communities, and those with no or little education – are at greatest risk [8]. Indian activists, policy-makers and scientists have called for urgent action to support survivors and prevent further violence [9].

What works to prevent violence against women and girls? Recent syntheses have documented promising approaches [10]. These include interventions with the legal and justice sectors, health providers, and communities. Community-based interventions broadly fall into two categories. The first consists largely of training to enable survivors and perpetrators to prevent violence or stop perpetrating it. The second involves wider community mobilisation to challenge norms and practices that perpetuate gender inequities and abet violence [10]. Such community mobilisation interventions usually engage a range of actors: women, men, the police, health workers, and political leaders. They use diverse strategies such as group-based dialogue, problem-solving and advocacy campaigns. Some community mobilisation interventions have succeeded in reducing violence, but the majority were tested in African contexts [11–13].

Most community interventions to prevent violence against women and girls tested in India have used group training and/or been conducted in peri-urban or urban settings with NGO facilitators [14–16]. A promising model developed by Mumbai non-government organisation SNEHA (Society for Nutrition, Education and Health Action) involves combining legal and counselling services for survivors, training to sensitise health providers and the police, and community mobilisation to shift norms and practices related to violence [17]. Though NFHS-4 data suggest violence against women and girls is more common in rural areas, there have been few evaluations of community mobilisation from rural India, and none with government-incentivised workers who could take such interventions to scale.

The National Health Mission’s c.900,000 Accredited Social Health Activists (ASHAs) are trained to assist women facing violence and support referrals to health, legal and police services [18]. In this study, we investigated the preliminary effects of community mobilisation through participatory learning and action groups facilitated by Accredited Social Health Activists (ASHAs), coupled with access to counselling to link survivors with services, in order to prevent violence against women and girls in Jharkhand, eastern India.

## Methods

### Objective and study design

We conducted baseline and endline cross-sectional surveys to assess the preliminary effects of a pilot community mobilisation intervention with participatory learning and action meetings facilitated by ASHAs to prevent violence against women and girls in West Singhbhum district, Jharkhand, between June 2016 and September 2017. We used a before-and-after design to establish the preliminary effects of the pilot intervention in preparation for a more rigorous, controlled evaluation. Such ‘piloting’ phases are recommended as part of the step-wise development and evaluation of complex interventions [19].

### Setting

In Jharkhand, girls and young women are highly vulnerable to trafficking, 44% of women aged 20–24 are married below the age of 18, and 35% of married women face spousal violence [5, 20]. Women in Jharkhand also face violence in the form of witch-hunting. Ethnographic studies suggest that witch-hunts are used to deal with social and economic misfortunes, acquire the accused’s land or property, or as punishment for women who challenge patriarchal norms [21]. Women accused of witchcraft are socially ostracised and sometimes assaulted or killed. India’s National Crime Records Bureau (NCRB) identified over 400 deaths linked to witchcraft accusations in Jharkhand over the past 15 years, the highest number in any Indian state [22].

Our intervention and cross-sectional surveys took place in 22 villages of Chakradharpur and Bandhgaon blocks, in West Singhbhum district. These villages were home to an estimated 16,110 people, 60% of whom were from *Adivasi* (indigenous) communities, and around 30% from Other Backward Castes [23]. Families mostly relied on single rain-fed paddy crops, food collected from the forest, and migratory trips for seasonal labour. Two-thirds of *Adivasi* village members were from the Ho *Adivasi* community [24]. Because women often contribute heavily to the household economy through income from agriculture, migration and the collection of forest produce, historians have described gender relations within Ho families as slightly more equitable than in other adjacent communities, and as "malleably patriarchal" [25].

### Intervention

Community mobilisation through Participatory Learning and Action (PLA) meetings with women’s groups is an approach that has been used extensively to improve maternal and newborn health in rural eastern India [25, 26]. In 2016, the National Health Mission endorsed the scale-up of PLA meetings through government-incentivised ASHAs and ASHA supervisors in ten Indian States. In Jharkhand, it currently supports PLA with over 30,000 women’s groups. Aside from facilitating PLA, ASHAs have their own government training in supporting survivors of violence. The training emphasises two strategies. The first is to build solidarity for survivors through *mahila mandals* (women’s groups), Village Health Nutrition and Sanitation Committee meetings, and *Gram Panchayat* (village council) meetings. The second is to respond to individual cases of violence by being alert to signs, providing emotional support, helping women facing severe violence find shelter, and linking women with health and legal services [18]. Building on this training, SNEHA and Ekjut partnered to pilot a community mobilisation intervention with ASHAs to prevent violence against women and girls. Prior to the intervention, six focus group discussions with married, unmarried, and elderly women and adolescent girls were conducted to inform the content and design of the intervention. Two ASHA supervisors provided substantial input into the intervention design, and were also trained in counselling to support survivors after the end of the project.

The intervention design had two guiding principles. The first is common to all approaches that emphasise the need for community participation: participation is a right, rather than simply being an option among many other behaviour change techniques [27, 28]. We assumed that women and other community members have the right to participate in decisions that will affect their lives. In complex social systems where the objective is to change multiple behaviours, behavioural theory remains critical, but externally-led formative research and design is often no match for local knowledge, ownership, and the ability to translate general intervention recommendations (e.g. discuss violence so it is no longer a private matter) into locally appropriate action. Our second guiding principle was informed by SNEHA’s experience: any community-based effort to raise the issue of violence needs to be coupled with the provision of counselling for survivors and activities to engage with legal, health and police services to meet their needs.

Before beginning activities in the community, Ekjut mapped local services available to survivors of violence and made contact with the local women’s police cell (*mahila thana*) where survivors can seek counselling and file First Information Reports (FIRs). A FIR is a document prepared by the police in response to information about a cognizable offence, i.e. one in which the police can arrest a person without warrant and start an investigation. With support from SNEHA, Ekjut also trained two counsellors and provided ASHAs with refresher training on local legal, health and police services. Finally, SNEHA conducted a sensitisation workshop with Superintendents of Police from across the State to help them understand their role in supporting survivors.

After this preliminary work, Ekjut re-activated 39 women’s groups in 22 villages which had previously taken part in a cycle of PLA meetings to improve maternal and newborn health. ASHAs in these villages were trained in three phases of three days each (nine days altogether), and then facilitated a four-phase PLA cycle with groups of 20–30 women each, in their own village, over 16 months (June 2016–September 2017).

Phase 1 included six meetings to discuss locally important forms of violence against women and girls. To aid discussion**,** ASHAs used picture cards depicting different forms of violence identified during qualitative formative work: early marriage and adolescent pregnancy; trafficking of girls and women; harassment in the workplace and in the street; witch-hunting; discrimination in food distribution or education; workload discrimination; dowry-related violence; second marriage; and domestic violence. During training and in discussions with groups, ASHAs were encouraged to emphasise patriarchy as an underlying cause of violence**.**

Phase 2 included three meetings in which group members analysed causes of the forms of violence they had prioritised through a mix of story-telling and games, then identified and prioritised locally feasible strategies to address them. In this second phase, the groups listened to and discussed a story that highlighted important social drivers of violence. For example, if a group prioritised domestic violence as a problem, the ASHA was asked to use her local knowledge to develop and tell a story that would include the following drivers: social norms that present women as inferior to men; re-marriage; seeing violence as a private matter; non-fulfilment of social and/or sexual expectations; alcohol use; large age gap between partners; past experience of abuse/violence and its acceptability; economic hardship. ASHAs were also encouraged to mention possible consequences of physical violence from a husband in their story (e.g. injuries, re-marriage, poor relationship with children). After listening to the story, group members discussed the causes and consequences of violence that resonated with their lives and those of others in the community.

Phase 3 consisted of five meetings focusing on forms of violence specific to Jharkhand (e.g. witch-hunting) and resources that could help strengthen the strategies prioritised by members (e.g. discussing the role of village leaders). In this phase, group members also reviewed the strategies they had been implementing and discussed their progress.

After 14 meetings, each group organised a larger community gathering involving other village residents, village leaders, and frontline health workers (Auxiliary Nurse Midwives and *Anganwadi* Workers). At this community meeting, group members shared the progress they had made and sought help from other community members to implement their strategies. Phase 4 consisted of a meeting at which the members assessed their achievements and decided on strategies that they would like to continue implementing in the future. During the course of the intervention, the intervention team referred several cases of women who were experiencing severe violence to the district welfare committee to seek their advice for further support. Table 1 summarises the sequence of meetings.

**Table 1:** Meeting plan

	Phase I: Identifying and Prioritising Problems
1	Introduction, community entry and consent (Street play and discussion)
2	Discussing the impact of power imbalances between men and women, boys and girls
3	Gender as a social construct (interactive discussion on men’s and women’s roles)
4	Violence throughout women’s lifecycle (storytelling)
5	Identifying problems related to violence against women and girls (role play on patriarchy)
6	Prioritising problems (voting using problem picture cards)
	Phase II: Analysing Problems and Exploring Solutions
7	Understanding the causes and effects of prioritised problems (storytelling)
8	Understanding barriers and opportunities for strategies identified by groups (bridge game)
9	Taking responsibilities for implementation and planning for a community meeting
	Phase III: Taking Action
10	Preventing trafficking of women and children (lion and goat game)
11	Preventing early marriages and early pregnancies (‘Pitthu’ game & Interactive discussion)
12	Preventing different forms of domestic violence (storytelling)
13	Preventing street harassment
14	Referrals for cases of violence and planning for a community meeting
	Phase IV– Evaluating Progress
15	COMMUNITY MEETING
16	Evaluation of activities by group members and dissemination

### Survey eligibility criteria and data collection

All women who were registered with the 39 women’s groups active in the 22 intervention villages were eligible to participate in the study. We attempted to interview all of these group members both before and after the intervention. Between April and July 2016, five female interviewers trained by Ekjut carried out a baseline survey with group members in their homes. Interviewers identified potential participants using self-help group registers, explained the purpose and process of the study to them, sought the participants’ verbal consent for interview, and entered their response on a smartphone. They made three attempts to interview each woman. The survey captured data on members’ socio-demographic characteristics as well as emotional violence by husbands and physical or emotional violence by others, including other family and community members. We selected indicators that we believed could be modified within the short timeframe of a pilot. The same cross-sectional survey was repeated from September to November 2017. Interviewers had three days of training and six days of practice before each survey. This was our first survey on violence in the area and we did not know whether women would be willing and able to discuss sexual violence, so we omitted questions on it in the surveys.

Following WHO recommendations, interviewers were trained in how to preserve women’s safety and confidentiality when asking questions about violence, how to provide emotional first aid if women were upset about a disclosure, and how to support women facing severe violence [29]. The remainder of the training focused on the use of smartphones and Dimagi’s electronic CommCare platform to collect data, followed by six days of practice. Interviewers had fortnightly review meetings during each survey. We used questions on violence from the NFHS-4 women’s survey, with additional ones related to ‘community violence’, defined as interpersonal violence perpetrated by individuals not intimately related to the victim [30]. This included witch-hunting, social boycott, and communal violence. The Ekjut team who trained ASHAs also regularly observed group meetings and collated short case studies on discussions and actions by group members.

### Statistical methods

We analysed data for all women who agreed to participate in the surveys, whether they had taken part in any intervention or not. We used descriptive statistics to report the proportion of women who found violence acceptable, who experienced emotional and physical violence, and who had sought help at baseline and endline. We used logistic regression models with random effects at the level of the cluster (village) to compute odds ratios for differences in outcomes between baseline and endline surveys. During preliminary analyses we identified differences in literacy, income regularity, and socio-economic deprivation among baseline and endline participants. We therefore report analyses unadjusted and adjusted for these variables. We also did a sub-group analysis that included only women who participated in both surveys.

### Ethical approval

We sought verbal consent from all study participants after reading out an information sheet and discussing questions that arose. The interviewer recorded whether consent was given or not on a smartphone. Our local ethics committee thought this process preferable to recording written consent via signature or thumbprint on paper because indigenous communities our study areas were often weary of signing paper documents due to past experiences of land and other property-related disputes. In order to assist with referrals to services as required, we constituted a project advisory group including representatives of local organisations working on violence and child trafficking.

## Results

### Response to the intervention

ASHAs successfully conducted monthly participatory learning and action meetings with 39 women’s groups in 22 villages of West Singhbhum district, Jharkhand, between June 2016 and September 2017. Each group prioritised three forms of violence against women and girls. Twenty-four (61%) of the 39 women’s groups prioritised domestic violence as a problem, 21 (53%) prioritised gender-based discrimination in workload, and 20 (51%) prioritised adolescent marriage and pregnancy. ASHAs then encouraged group members to think about collective and individual strategies to address their prioritised problems. All 39 groups committed to providing counselling to families of survivors, encouraging them to report violence to community health workers and local leaders, and raising awareness of the consequences of violence against women and girls with help from service providers and governance leaders. Twenty-one groups organised community campaigns and street plays against early marriage, and also engaged with match-makers to dissuade them from recommending early marriage. Nineteen groups committed to ensuring participation of adolescents in discussions about the prevention and consequences of early marriage in all community meetings, including Village Health and Nutrition Days. Finally, seven groups committed to seeking help from the police when women faced street harassment or witch-branding. In addition to the participatory learning and action meetings, two counsellors facilitated referrals or discussions with local village leaders (*munda*s) for 27 women, two women sought further assistance from the police, and 58 women received counselling. The Child Welfare Committee, community health workers and PLA group members estimated that their efforts prevented the trafficking of 15 girls.

### Preliminary intervention effects

The survey team interviewed 59% (679/1149) of women registered with the 39 women’s groups at baseline, and 63% (861/1371) at endline. Refusal rates were low: one woman did not consent to participate at baseline and two at endline. 55% (373/679) of women who had participated in the baseline were also interviewed at endline. We excluded 64 cases from endline analyses on the acceptability of violence, as the survey team found that one out of 11 interviewers had misunderstood these survey questions.

Table 2 shows participants’ characteristics. More women were interviewed at endline than baseline. In both baseline and endline surveys, over 60% of participants were from *Adivasi* communities (adjusted *p*-value for difference between surveys, *p* = 0.522). There were more literate women at endline than baseline (47% vs 33%, adjusted *p* < 0.001). Similarly, fewer women were socio-economically disadvantaged – defined as being *Adivasi* and having no bank account – at endline than baseline (23% vs 15%, adjusted *p* < 0.001). We therefore report results for all baseline and endline participants, adjusting for key socio-economic variables (literacy, income regularity and socio-economic deprivation), and then for the sub-group of 373 women who completed both surveys.

**Table 2:** Participants’ characteristics

	BASELINE		ENDLINE		***P***^***1***^
	***n***	%	***n***	%	
Tribe or Caste					
*Adivasi* (Scheduled Tribe)	445	65.5	574	63.5	
Scheduled Caste	41	6.0	43	5.0	
Other Backward Class	192	28.3	269	31.2	
None of the above	1	0.1	2	0.2	0.522
Literacy					
Cannot read or with difficulty	452	66.6	458	53.2	
Can read	227	33.4	403	46.8	< 0.001
Has a regular source of income^**3**^					
Yes	292	43.0	295	34.3	
No	387	57.0	566	65.7	< 0.001
Occupation					
Salaried job	16	2.4	29	3.4	
Farming	205	30.2	162	18.8	
Labourer	203	29.9	353	41.0	
Housewife	248	36.5	283	32.9	
Student	7	1.0	16	1.9	
Small business	0	0.0	18	2.1	< 0.001
Card ownership					
Above Poverty Line (APL)	19	2.8	1	0.1	
Antyodaya	219	32.2	202	23.5	
Below Poverty Line (BPL)	276	40.6	603	70.0	
None of the above	165	24.3	55	6.4	< 0.001
Socio-economically disadvantaged^**4**^					
Yes	155	22.8	129	15.0	
No	524	77.2	732	85.0	< 0.001
Family type					
Nuclear	440	64.0	553	64.2	
Joint	237	34.9	305	35.4	
Extended	2	0.3	3	0.3	0.960
Marital status					
Married	532	78.3	670	77.8	
Unmarried	44	6.5	52	6.0	
Widow	103	15.2	135	15.7	
Divorced/separated	0	0	4	0.5	0.341
Total respondents	**679**	**100**	**861**	**100**	

^1^*P* values for differences in participants’ characteristics between baseline and endline derived from chi-squared tests for categorical variables and t-tests for continuous variables

^3^ Defined as daily wage rather than seasonal or other types of intermittent wage

^4^ Defined as being *Adivasi* and not having a bank account

#### Acceptability of violence

Table 3 describes the acceptability of violence before and after the intervention. We found small differences between baseline and endline surveys, with fewer women reporting that violence was acceptable if a woman went out without telling anyone (8.5 vs 4.9%), neglected the house or children (9.6 vs 6.5%, or if her husband suspected infidelity (10.6 vs 7.3%). 82.9% of women reported that violence was unacceptable in all seven scenarios presented to them at endline, compared to 74.3% at baseline (adjusted *p* < 0.001).

**Table 3:** Acceptability of physical violence (all participants)

		Baseline		Endline		Unadjusted Odds Ratio (95% CI) ^**1**^	P	Adjusted Odds Ratio (95% CI) ^**2**^	*P*
		*n*	%^4^	n^3^	%^4^				
Physical violence against a woman is acceptable if:									
She goes out without telling anyone		58	8.5	39	4.9	0.51 (0.33–0.79)	0.003	0.51 (0.31–0.81)	0.005
She neglects the house or children		65	9.6	52	6.5	0.64 (0.43–0.96)	0.030	0.62 (0.41–0.97)	0.038
She argues with her husband or family member		36	5.3	45	5.6	1.07 (0.67–1.70)	0.781	1.12 (0.67–1.86)	0.660
She refuses to have sex with her husband		18	2.6	20	2.5	0.99 (0.51–1.93)	0.975	0.95 (0.46–1.97)	0.886
She does not cook food properly		35	5.1	30	3.8	0.71 (0.42–1.18)	0.188	0.74 (0.42–1.31)	0.307
Her husband suspects her of being unfaithful		72	10.6	58	7.3	0.57 (0.39–0.84)	0.004	0.60 (0.39–0.90)	0.015
She becomes disrespectful of her husband/in-laws		42	6.2	42	5.3	0.83 (0.53–1.32)	0.439	0.96 (0.58–1.58)	0.880
Violence is unacceptable in all of the above situations		504	74.3	661	82.9	1.87 (1.42–2.46)	< 0.001	1.87 (1.39–2.52)	< 0.001
Total respondents		**679**	**100**	**797**	**1000**	–	–	–	

^1^ Adjusted for clustering by village with random effect term only

^2^ Adjusted for literacy, income regularity, socio-economic disadvantage and clustering by village

^3^ We excluded 64 cases from an interviewer who had misunderstood questions on the acceptability of violence

^4^ Proportions may not add to up 100 due to missing data

#### Past year emotional violence from husbands and other family members

Table 4 describes the prevalence of past year emotional violence from husbands before and after the intervention. The proportion of women who experienced emotional violence from husbands in the past 12 months decreased from 67.3 to 56.2% (adjusted *p* < 0.001). The proportion of women seeking help for emotional violence from husbands increased, from 28.3 to 45.7% (adjusted *p* < 0.001). Help-seeking from within the family, in-laws, or from within the village all increased, but most help-seeking remained within the family (42.5% at endline) or village (42.3% at endline). Less than 3% of women sought help from frontline workers, health facilities, panchayat members, the police or legal aid cell.

**Table 4:** Emotional violence from husbands in the past year (all participants)

	BASELINE		ENDLINE		UNADJUSTED ODDS RATIO (95% CI) ^1^	*P*	ADJUSTED ODDS RATIO (95% CI) ^2^	*P*
	*n*	%^4^	*n*	%^4^				
Past year emotional violence by husband								
Yes	457	67.3	484	56.2	0.56 (0.45–0.70)	< 0.001	0.55 (0.43–0.71)	< 0.001
No	222	32.7	377	43.8				
Total respondents	679	100	861	100				
Help-seeking for past year emotional violence by husband^3^								
Yes	129	28.3	221	45.7	2.05 (1.58–2.81)	< 0.001	2.19 (1.51–3.17)	< 0.001
No	310	67.8	263	54.3				
Total respondents	457	100	484	100				
Persons from whom respondents sought help ^3^								
Own family	49	38.0	94	42.5	1.34 (0.81–2.22)	0.248	1.07 (0.57–2.03)	0.824
In-laws	14	10.8	44	19.9	1.91 (0.97–3.80)	0.062	2.18 (0.91–5.22)	0.081
Within village	34	25.7	94	42.3	2.29 (1.34–3.91)	0.002	2.72 (1.38–5.37)	0.004
Total respondents	129	100	221	100				

^1^ Adjusted for clustering by village with random effect term only

^2^ Adjusted for literacy, income regularity, socio-economic disadvantage and clustering by village

^3^ Respondents who experienced emotional violence from husband in the past year only

^4^ Proportions may not add to up 100 due to missing data

Table 5 describes the prevalence of past year emotional and physical violence from family members others than husbands. The prevalence of past year emotional violence from family members decreased, from 66.0 to 50.1% (adjusted *p* < 0.001), as did the prevalence of past year physical violence from family, from 27 to 12% (adjusted *p* < 0.001). The proportion of women seeking help for violence from family members increased from 35.2 to 66.8% (*p* < 0.001). Over half sought help from within the family (15.7% at endline), or from within the village (35.5% at endline). Less than 5% sought help from frontline workers, health facilities, panchayat members, the legal aid cell or police. We did not collect data on physical violence among husbands at baseline and are unable to assess changes in this. The prevalence of past year physical violence from husbands was high (23.8%, or 205/861) at endline.

Although we did not collect data on the prevalence of sexual violence, qualitative feedback from ASHAs and group members indicated that they intervened in some cases of sexual violence. We include a case study on sexual violence below as supportive evidence, with people’s names changed for anonymity.

**Table 5:** Past year emotional and physical violence by family members other than husbands (all participants)

	Baseline		Endline		Unadjusted Odds Ratio (95% CI) ^**1**^	*P*	Adjusted Odds Ratio (95% CI) ^2^	*P*
	*n*	%^3^	*n*	%^3^				
Past year emotional violence by other family members								
Yes	448	66.0	431	50.1	0.44 (0.35–0.56)	< 0.001	0.41 (0.32–0.53)	< 0.001
No	231	34.0	430	49.9				
Total respondents	679	100	861	100				
Past year physical violence by other family members								
Yes	184	27.1	104	12.1	0.34 (0.26–0.45)	< 0.001	0.36 (0.26–0.50)	< 0.001
No	495	72.9	757	87.9				
Total respondents	679	100	861	100				
Perpetrators of emotional and physical violence (other family members)								
Father-in-law	126	27.7	137	30.8	1.20 (0.88–1.65)	0.252	0.89 (0.62–1.28)	0.544
Brother-in-law	75	16.5	111	24.9	1.85 (1.30–2.64)	0.001	1.96 (1.31–2.91)	0.001
Other in-law	287	63.1	125	28.1	0.17 (0.12–0.23)	< 0.001	0.21 (0.14–0.30)	< 0.001
Other relative	202	44.4	102	22.9	0.36 (0.26–0.50)	< 0.001	0.42 (0.29–0.60)	< 0.001
Total respondents	455	100	445	100				
Help-seeking for violence by other family members								
Yes	160	35.2	293	65.8	4.58 (3.25–6.45)	< 0.001	4.45 (3.04–6.52)	< 0.001
No	463	67.9	190	35.5				
Total respondents	295	64.8	152	34.2				
Persons from whom respondents sought help								
Own family	46	28.7	46	15.7	0.29 (0.17–0.51)	< 0.001	0.27 (0.14–0.52)	< 0.001
In-laws	24	15.0	14	4.8	0.16 (0.07–0.38)	< 0.001	0.13 (0.05–0.35)	0.001
Within village	54	33.7	104	35.5	1.17 (0.73–1.87)	0.512	1.36 (0.77–2.41)	0.286
Total respondents	160	100	293	100				

^1^ Adjusted for clustering by village with random effect term only

^2^ Adjusted for literacy, income regularity, socio-economic deprivation and clustering by village

^**3**^ Proportions may not add up to 100 due to missing data

^4^ We asked about husbands as a source of support at baseline but not endline. Data are presented without including husbands in ‘own family’

#### Case study: a ‘second marriage’ (*dusri shaadi*)

Seema lived with her husband and five children. She did agricultural work to meet household expenses. Her husband Vinod did not work or take care of their children. He often drank, kept fighting with Seema, and forced her to have sex with him. Even during illness, he forced himself on her and refused to help her go for a check-up, which made her even sicker. During the sixth month of pregnancy, Vinod forced Seema to have sex with him. After delivery, Seema’s health worsened so much that she was unable to walk normally for a whole year. Because of this, Vinod married another woman, which was allowed by customary law, but made him care less for Seema and their children. Their four-year-old child died. Because of her own health problems, Seema was not able to look after her children or go to work, and was still not receiving any help from her husband. One day, a women’s group member told Seema that the ASHA was conducting meetings once a month where issues like ‘second marriages’ and violence against women were discussed, so the ASHA might be able to help her. The next day, Seema went to the ASHA, who invited her to the next meeting. The ASHA said that she would invite Seema’s husband and the second wife too. In the meeting, the ASHA, with the help of community members, performed a role play showing how a woman was beaten and forced to have sex by her husband. After attending the meeting, Vinod said that he kept thinking about the role play. He realised that he had been neglecting his wife, as shown in the play. The second wife and Vinod started liking the ASHA’s meetings and attending them. The ASHA met with Vinod separately to counsel him on how he could take care of his children, and the local village headman also asked him to take responsibility for his children.

#### Community violence

Table 6 describes the prevalence of community violence, which included witch-hunting, social boycott, communal violence, or being prevented from accessing public facilities or common resources. We found no evidence of change in this type of violence (15.0% at baseline; 11.3% at endline, adjusted *p* = 0.226) or help-seeking for it (63.7% at baseline and 64.9% at endline, adjusted *p =* 0.159).

**Table 6:** Experiences of, and help-seeking for, community violence

	Baseline		Endline		Unadjusted Odds Ratio (95% CI) ^1^	*P*	Adjusted Odds Ratio (95% CI) ^2^	*P*
	*n*	%	*n*	%				
Past year experience of community violence*								
Yes	102	15.0	97	11.3	0.76 (0.56–1.04)	0.085	0.81 (0.58–1.14)	0.226
No	576	84.8	755	87.7				
Total respondents	679	100	861	100				
Help-seeking for community violence								
Yes	65	63.7	63	64.9	1.28 (0.64–2.55)	0.488	1.77 (0.80–3.93)	0.159
No	37	36.3	34	35.0				
Total respondents	102	100	97	100				

* The following examples were given: witch-hunting, communal violence, social boycott, being prevented from accessing public facilities or common resources, being subjected to a community**-**imposed penalty

^1^ Adjusted for clustering by village with random effect term only

^2^ Adjusted for literacy, income regularity, socio-economic disadvantage and clustering by village

#### Sub-group analysis of effects on women who participated in both baseline and endline surveys

As shown in Supplementary Table 1, we found no differences between women who participated in both baseline and endline surveys and those who participated in the overall baseline. As in the broader sample, more women reported that violence against women was unacceptable in all seven scenarios presented to them at endline (84%) compared to baseline (72%, unadjusted *p* < 0.001). Past year emotional violence from husbands decreased from 72 to 57% (unadjusted *p* < 0.001), and help-seeking increased from 29 to 43% (unadjusted *p* = 0.035). Half of women (51%) sought help within their own family and 34% from within the village. Again, as in the broader sample, the prevalence of past year emotional violence by family members decreased substantially, from 68 to 44%, as did the prevalence of past year physical violence by family members, from 25 to 12% (*p* < 0.001). Fewer women in the sub-sample had experienced community violence at endline (9%) than at baseline (15%, *p* = 0.025).

## Discussion

Our study is the first to explore the preliminary effects of a community mobilisation intervention to prevent violence against women and girls with ASHAs in rural India. The intervention used an approach (participatory learning and action) endorsed by the National Health Mission. Our data suggest that the acceptability of violence against women decreased among group members exposed to the intervention. Members were also more likely to discuss violence and seek help from family or within the village after the intervention. These changes may have led to actual reductions in violence, although a larger, controlled evaluation is clearly required to provide more rigorous evidence of impact. Our preliminary conclusions are supported by the fact that changes were also reported by women who completed both baseline and endline surveys, a proxy for greater potential exposure to the intervention. Our process data suggest that changes in emotional violence were probably not solely the result of PLA meetings, and were also most likely influenced by ASHA supervisors actively helping survivors and counsellors, and following up to provide individual women with support either through the district welfare committees, linkages with the police or through local self-governance. These pathways merit further exploration in future evaluations.

The prevalence of past year emotional violence from husbands (67%) was much higher than that found in the National Family Health Survey (NFHS-4) for rural Jharkhand (10%) [5]. This may reflect the fact that we asked about more types of emotional violence than NFHS-4, which only considers four manifestations of emotional violence. Our estimate is closer to that of Babu et al. (52%), who used a set of questions similar to ours [31].

Interestingly, the greatest reductions in emotional violence from family members were among ‘other relatives’ and ‘other in-laws’, which may point to mothers-in-law. Qualitative feedback from ASHAs and group members indicate that members were a source of help for emotional and physical violence within the family, as were husbands when other relatives were perpetrators. Women’s preference for seeking help from trusted relatives or friends before turning to health services or the police is well documented in India and elsewhere [5, 32]. Future surveys could collect finer-grained data on perpetrators of violence within the family and sources of help within the family and village to guide future interventions.

While help-seeking appeared to increase overall after our intervention, less than 5% of women sought help from health professionals, the police or legal aid cell. Sensitising local governance systems through *munda*s (headmen), investing in public services to make them more responsive to survivors, and creating better linkages between groups, ASHAs, and services might help to increase help-seeking further. An important finding from our collation of stories from groups is that some group members, ASHAs, and village leaders took direct action by confronting perpetrators. While this was clearly welcome in many instances, there is potential for groups to turn to vigilantism and bypass services, at least in the short term. This would need to be monitored as a potential consequence of the intervention [33].

Our study had some limitations. First, we were not able to enrol a control group or use randomisation, both of which would have greatly enhanced our ability to obtain an unbiased estimate of the intervention’s effects. Our study design makes it impossible to rule out selection bias or our results being caused by secular changes in the prevalence of violence due to events unrelated to the intervention. Nevertheless, we achieved our aim of piloting the intervention and assessing its preliminary effects in preparation for a more rigorous, controlled evaluation. Second, we only collected data on physical violence from husbands, but not on sexual violence. This means that we are likely to have underestimated the extent of violence in our study area, and are unable to comment on the intervention’s effects on the most common form of violence against women. Third, we were only able to find and interview 59% of women’s group members at baseline and 63% of members at endline. Women and men often migrate for work in brick kilns or cities between December and June, and it is possible that some group members had not returned by the time the baseline began in April 2016. In addition, some women heard about the content of the interviews from others and refused to be approached entirely, even prior to sharing information about the study. It is possible that women who were travelling or refused to participate in the study were at greatest risk of violence, and would have responded less well to the intervention. Fourth, changes in acceptability, violence and help-seeking observed between baseline and endline surveys may have been caused by the fact that respondents in the endline survey were somewhat better off socio-economically than those in the baseline. Finally, we cannot rule out the possibility of social desirability bias in women’s reports, as they attended meetings where violence was explicitly condemned. This community mobilisation approach through participatory learning and action with ASHAs needs further evaluation on a more complete set of violence outcomes, through a controlled comparison to minimize the effects of selection bias and secular change and with more complete qualitative data collection to better understand pathways to violence prevention.

What do our findings mean for policy? While our data provide some justification for further testing participatory learning and action for violence prevention with ASHAs, we should be cautious about placing the task of identifying and supporting women facing violence solely on the shoulders of ASHAs, given their multiple activities. Instead, the literature on community mobilisation and SNEHA’s experience suggest the need for a more comprehensive approach to reducing violence against women, with interventions at multiple levels [10, 17]. In the community, ASHAs could be supported by women’s group members or community volunteers to help identify and support women facing violence. Such an approach would also need to be complemented by support for counselling centres and training to enable health, legal and police services to respond appropriately to survivors. Another noteworthy stream of intervention is emerging beyond the health sector, through the National Rural Livelihood Mission’s Social Action Committees (SAC). SAC were initially developed as part of long-term poverty-alleviation programmes with self-help groups in Andhra Pradesh and Telangana. They provide informal counselling to women who face violence, offer mediation using principles of restorative justice, and refer cases to the police or formal justice system when violence is severe or mediation is impossible [34]. SAC draw inspiration from earlier, now largely disbanded government-supported *Mahila Samakhya* (groups advocating for women’s equality) and *Nari Adalats* (informal women’s courts) [35]. To our knowledge, there have been no *controlled* evaluations of the effects of community mobilisation using principles of participatory learning and action or contemporary SAC on violence against women and girls in rural areas. Both types of intervention could be important focal areas for future research.

## Conclusion

Community mobilisation through participatory learning and action meetings with ASHAs is an acceptable approach to raise the issue of violence against women in rural communities of Jharkhand. Our pilot found promising preliminary evidence of reductions in the acceptability of violence, experiences of past year emotional and physical violence, as well as help-seeking. As the National Health Mission continues to refine the portfolio of community interventions offered by ASHAs, the inclusion of community mobilisation to prevent violence could benefit a large number of women and girls in rural areas, as part of a comprehensive approach to violence prevention.

## Supplementary information

**Additional file 1: Table S1.** Comparison of the characteristics of women who participated in both baseline and endline surveys.

**Additional file 2.** Study dataset.

**Additional file 3.** Questionnaire.

## Data Availability

All data generated or analysed during this study are included in this published article and its supplementary information files 1 (the dataset) and 2 (questionnaire).

## Abbreviations

ASHA: Accredited Social Health Activist
FIR: First Information Report
NFHS: National Family Health Survey
NHM: National Health Mission
PLA: Participation Learning and Action
SNEHA: Society for Nutrition, Education and Health Action
