# Supplementary material for: Community mobilisation to prevent violence against women and girls in eastern India through participatory learning and action with women’s groups facilitated by accredited social health activists: a before-and-after pilot study
Source: BMC Int Health Hum Rights. 2020 Mar 25;20:6. doi: 10.1186/s12914-020-00224-0 (PMC7093987; doi:10.1186/s12914-020-00224-0)
Supplement: Supplementary file 1 — Additional file 1: Table S1. Comparison of the characteristics of women who participated in both baseline and endline surveys. [file 12914_2020_224_MOESM1_ESM.docx]

**SUPPLEMENTARY FILE**

**Supplementary Table 1:**

Comparison of the characteristics of women who participated in both baseline and endline surveys

|  | **BASELINE** | | **PARTICIPATED IN BASELINE AND ENDLINE** | | ***P ^2^*** |
| --- | --- | --- | --- | --- | --- |
|  | n | %^1^ | n | %^1^ |  |
| **Tribe or Caste** |  |  |  |  |  |
| *Adivasi* (Scheduled Tribe) | 445 | 65.5 | 253 | **100** |  |
| Scheduled Caste | 41 | 6.04 | 24 |  |  |
| Other Backward Class | 192 | 28.3 | 95 | 67.8 |  |
| None of the above | 1 | 0.1 | 1 | 6.4 | 0.261 |
| **Literacy** |  |  |  | 25.4 |  |
| Cannot read or with difficulty | 452 | 66.6 | 248 | 0.3 |  |
| Can read | 227 | 33.4 | 125 |  | 0.961 |
| **Has a regular source of income^3^** |  |  |  | 66.5 |  |
| Yes | 292 | 43.0 | 163 | 33.5 |  |
| No | 387 | 57.0 | 210 |  | 0.686 |
| **Occupation** |  |  |  | 43.7 |  |
| Salaried job | 16 | 2.4 | 9 | 56.3 |  |
| Farming | 205 | 30.2 | 116 |  |  |
| Labourer | 203 | 29.9 | 106 | 2.4 |  |
| Housewife | 248 | 36.5 | 139 | 31.1 |  |
| Student | 7 | 1.0 | 3 | 28.4 | 0.848 |
| **Card ownership** |  |  |  | 37.3 |  |
| Above Poverty Line (APL) | 19 | 2.8 | 11 | 0.8 |  |
| *Antyodaya* (Poorest) | 219 | 32.2 | 103 |  |  |
| Below Poverty Line (BPL) | 276 | 40.6 | 168 | 2.9 |  |
| None of the above | 165 | 24.3 | 91 | 27.6 | 0.023 |
| **Socio-economically disadvantaged^4^** |  |  |  | 45.0 |  |
| Yes | 155 | 22.8 | 87 | 24.4 |  |
| No | 524 | 77.2 | 286 |  | 0.734 |
| **Family type** |  |  |  | 23.3 |  |
| Nuclear | 440 | 64.0 | 245 | 76.7 |  |
| Joint | 237 | 34.9 | 126 |  |  |
| Extended | 2 | 0.3 | 2 | 65.7 | 0.361 |
| **Marital status** |  |  |  | 33.8 |  |
| Married | 532 | 78.3 | 304 | 0.5 |  |
| Unmarried | 44 | 6.5 | 21 |  |  |
| Widow | 103 | 15.2 | 48 | 81.5 |  |
| Divorced/separated | 0 | 0 | 0 | 5.6 | 0.088 |
| **Total respondents** | **679** | **100** | **373** | **100** |  |

^1^ Proportions may not add to up 100, due to missing data.

^2^ P values for differences in the characteristics of women who participated in the baseline and in both baseline and

endline surveys, derived from chi-squared test for categorical variables and t-tests for continuous variables
